# Expression of genes in the skeletal muscle of individuals with cachexia/sarcopenia: A systematic review

**DOI:** 10.1371/journal.pone.0222345

**Published:** 2019-09-09

**Authors:** Cecily A. Byrne, Amy T. McNeil, Timothy J. Koh, Amelia F. Brunskill, Giamila Fantuzzi

**Affiliations:** 1 University of Illinois at Chicago, College of Applied Health Sciences, Department of Kinesiology and Nutrition, Chicago, IL, United States of America; 2 University of Illinois at Chicago, Library of the Health Sciences, Chicago, IL, United States of America; SERGAS and IDIS, SPAIN

## Abstract

**Background:**

Cachexia occurs in individuals affected by chronic diseases in which systemic inflammation leads to fatigue, debilitation, decreased physical activity and sarcopenia. The pathogenesis of cachexia-associated sarcopenia is not fully understood.

**Objectives:**

The aim of this systematic review is to summarize the current evidence on genes expressed in the skeletal muscles of humans with chronic disease-associated cachexia and/or sarcopenia (cases) compared to controls and to assess the strength of such evidence.

**Methods:**

We searched PubMed, EMBASE and CINAHL using three concepts: cachexia/sarcopenia and associated symptoms, gene expression, and skeletal muscle.

**Results:**

Eighteen genes were studied in at least three research articles, for a total of 27 articles analyzed in this review. Participants were approximately 60 years of age and majority male; sample size was highly variable. Use of comparison groups, matching criteria, muscle biopsy location, and definitions of cachexia and sarcopenia were not homogenous. None of the studies fulfilled all four criteria used to assess the quality of molecular analysis, with only one study powered on the outcome of gene expression. *FOXO1* was the only gene significantly increased in cases versus healthy controls. No study found a significant decrease in expression of genes involved in autophagy, apoptosis or inflammation in cases versus controls. Inconsistent or non-significant findings were reported for genes involved in protein degradation, muscle differentiation/growth, insulin/insulin growth factor-1 or mitochondrial transcription.

**Conclusion:**

Currently available evidence on gene expression in the skeletal muscles of humans with chronic disease-associated cachexia and/or sarcopenia is not powered appropriately and is not homogenous; therefore, it is difficult to compare results across studies and diseases.

## Introduction

Cachexia is a complex metabolic condition that occurs in individuals affected by chronic diseases such as cancer, chronic obstructive pulmonary disease (COPD) and chronic kidney disease (CKD) [1]. Sarcopenia is the most important phenotypic characteristic of cachexia [2]. Clinical definitions of cachexia and sarcopenia are updated as knowledge of these conditions progresses. Currently, cachexia is defined as loss of muscle with or without the loss of fat mass in the presence of an underlying illness ]1]. The most recent definition of sarcopenia uses low muscle strength as the primary criterion, with low muscle quantity and/or quality as a confirming parameter and degree of physical performance as a measure of severity [3]. Despite the development of distinct definitions for sarcopenia and cachexia, a ‘salad of terms’, including malnutrition, wasting or involuntary weight loss in the context of cachexia as well as muscle atrophy, weakness, frailty or wasting in the context of sarcopenia, is often used interchangeably in the scientific literature, generating confusion and difficulty in comparing and interpreting studies [1, 4].

While cachexia is always associated with an underlying illness, aging, inflammation as well as lack of physical activity or adequate nutrition can all cause or contribute to development of sarcopenia [4]. There are currently no effective treatments for cachexia or sarcopenia. Research aims at developing targeted interventions through a better understanding of the pathogenesis of muscle loss in individuals with chronic disease-associated cachexia or sarcopenia, including identification of genes that act as key mediators [5, 6]. Mechanisms driving muscle loss may include increased degradation of skeletal muscle protein, decreased protein synthesis, and abnormalities in apoptosis or autophagy [7]. Most of this work has been done in animal models and using in vitro approaches. For instance, animal models of muscle atrophy demonstrated a consistent increase in the gene expression of *FBXO32 (ATROGIN1)* and *TRIM63 (MURF1)* [8–11], which are part of the ubiquitin proteasome pathway involved in protein degradation. However, these findings have not been consistently confirmed in human studies [5, 6, 12–17]. Several reasons may explain why evidence from animal models and in vitro studies does not always translate to humans, including difficulty in developing appropriate and relevant animal models [2] as well as lack of standardization of human studies [6].

Cachexia and sarcopenia occur across the spectrum of chronic disease, but individual studies usually focus on only one or a few conditions. Studies in this field tend to be small and to investigate only a few genes or molecular pathways at a time. Evidence on gene expression in the skeletal muscle of individuals with cachexia and/or sarcopenia across different chronic diseases has not been systematically analyzed and evaluated to date; therefore, we don’t have an overall understanding of the current knowledge in the field. The aim of this systematic review is to summarize the current evidence on genes expressed in the skeletal muscles of humans with chronic disease-associated cachexia and/or sarcopenia (cases) compared to controls and to assess the strength of such evidence.

## Methodology

This systematic review was originally developed to summarize and evaluate the evidence for gene and protein expression in skeletal muscles of individuals with chronic disease-associated cachexia and/or sarcopenia. Hence, the search strategy focused on both gene and protein expression. Although it makes sense to simultaneously analyze gene and protein expression when focusing on a few genes or proteins, the systematic search returned 151 genes and 63 proteins, many of which were analyzed under different post-translational variants. Moreover, there was only a partial overlap between the genes and the proteins studied. Therefore, the investigators decided to focus the present review on gene expression and to analyze protein expression separately.

This systematic review was registered with the International Prospective Register for Systematic Reviews (PROSPERO) under protocol number CRD4201809361.

### Search strategy

The databases PubMed, EMBASE and CINAHL were searched on March 23, 2018 using controlled vocabulary and key words for three concepts: cachexia/sarcopenia and associated symptoms, gene/protein expression, and skeletal muscle. The search was not restricted in regard to publication period but excluded any article that was not a peer-reviewed primary publication. The search strategies for these databases are presented in S1 File.

**Fig 1:**
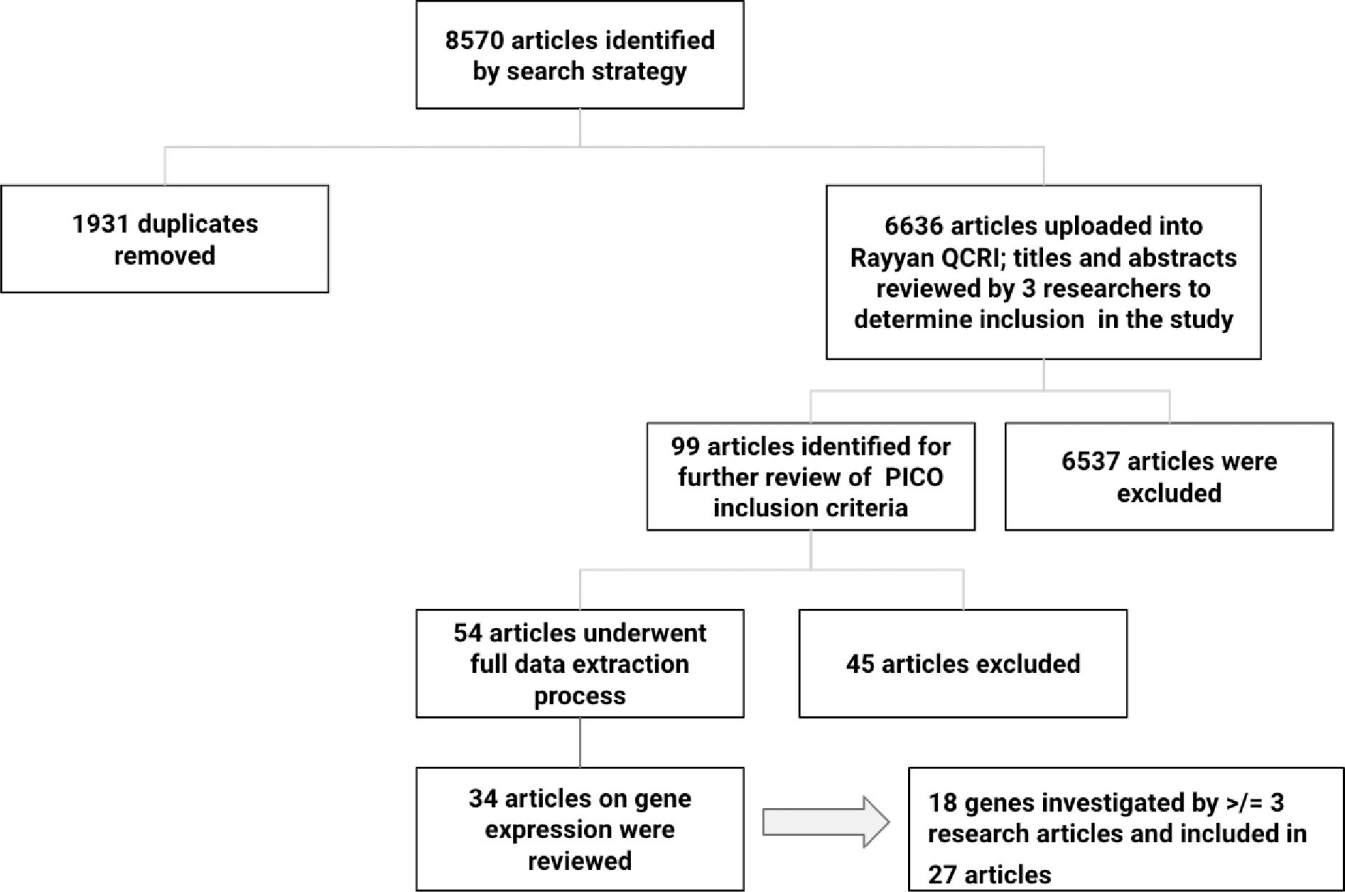
Flow chart indicating the process for article exclusion and derivation of the final 27 articles for inclusion in the systematic review on gene expression.

Fig 1 depicts the process of article exclusion and derivation of articles and genes for the systematic review. The search strategy returned 8570 articles of which 1932 were duplicates. Upon review of 6636 abstracts by three researchers (GF, CB, AM), 99 articles were identified for further review of PICO (population, intervention, control, outcome) criteria. Observational studies of adults ≧18 years of age that met the following PICO criteria were included: 1) Population: individuals with chronic disease-associated cachexia and/or sarcopenia (cases); 2) Intervention: skeletal muscle biopsy; 3) Control: healthy subjects or individuals with chronic disease but no cachexia/sarcopenia; 4) Outcome: gene expression. Reference lists of included articles were hand-searched for identification of additional relevant articles.

Articles were excluded from the review if the wrong population (e.g., sarcopenia of aging, weight loss in obesity, malnutrition) or outcome (microRNAs, single nucleotde polymorphisms) was studied, if the experimental approach focused only on animal or in vitro studies, if the publication was not a peer-reviewed primary article (e.g., review article, thesis or poster session), or if it was published in a language other than English. Fifty-four articles underwent the process of full data extraction, and 34 articles were included in the systematic review of gene expression in skeletal muscle of individuals with chronic disease-associated cachexia and/or sarcopenia. Upon reviewing the results of the 151 genes investigated in the 34 articles, the researchers summarized and evaluated evidence related to expression of those genes investigated in at least three independent research articles. Eighteen genes were studied in at least three research articles, for a total of 27 articles (see Table 1). These 18 genes and 27 articles are the focus of this systematic review (see Table 2).

**Table 1:** Summary of 27 studies investigating the 18 genes included in the systematic review.

Author/Year/ Disease	Subjects: Number and demographics	Cachexia/Sarcopenia^a^	Muscle/ Technique	Gene Expression
Aversa/2016/ Cancer	29 cancer cases (stratified as 12 CC and 17 NCC); 11 controls (abdominal surgery for non-neoplastic diseases). Age: 68±2 cases; 63±4 controls. Sex: M/F 17/12 cases; 6/5 controls. Race/Ethnicity not reported. Matched by age and sex.	Cachexia: **Weight loss >5% in 6 months**; Body weight; BMI. Sarcopenia: FFMI by BIA without set threshold.	Rectus abdominusqPCR	*BECN1*:NS *BNIP3*: NS *MAP1LC3B*: increased in CC vs controls; NS in CC vs NCC and NCC vs controls *SQSTM1*: NS
Bonetto/2013/ Gastric Cancer	16 cancer cases (sub-analysis based on weight loss); 6 controls (abdominal surgery for non-neoplastic diseases); Age: 64±3 cases; 62±6 controls. Sex/Race/Ethnicity not reported Matching criteria not reported.	Cachexia: Weight loss >5% from UBW; BMI. Sarcopenia: Not assessed.	Rectus abdominusqPCR, sqPCR	*FBXO32*: decreased in cases vs controls irrespective of weight loss by sqPCR; NS by qPCR *IGF-1*: decreased in cases vs controls irrespective of weight loss *MSTN*: decreased in cases vs controls irrespective of weight loss *TRIM63*: NS
Bossola/ 2001/ Gastric Cancer	20 gastric cancer cases (sub-analysis based on weight loss); 10 controls (abdominal surgery for non-neoplastic diseases). Age: 62±18 cases; 62±14 controls. Sex: M/F 11/9 cases; 6/4 controls. Race/Ethnicity not reported. Matching criteria not reported.	Cachexia: Weight loss >5% from UBW; Body weight; BMI. Sarcopenia: Not assessed.	Rectus abdominusNorthern blot	*UBIQUITIN* increased in cases vs controls irrespective of weight loss
Bossola/2002/ ESRD on HD	8 ESRD on HD cases; 6 non-ESRD controls (selective laparotomy for various non-septic conditions Age: 43±3 cases; 42±3 controls. Sex: M/F 4/4 cases; 3/3 controls. Race/Ethnicity not reported. Matching criteria not reported.	Cachexia: **Weight loss >5% in 6 months**; Body weight; BMI. Sarcopenia: Not assessed.	Rectus abdominusNorthern blot	*UBIQUITIN* NS
Bossola/2003/ Gastric cancer	23 gastric cancer cases; 14 controls (surgery for benign abdominal diseases). Age: 59±16 cases; 61±12controls. Sex: M/F 14/9 cases; 9/5 controls; Race/Ethnicity not reported. Matching criteria not reported.	Cachexia: Weight loss >5% from UBW; Body weight; BMI. Sarcopenia: Not assessed.	Rectus abdominusNorthern blot	*UBIQUITIN* increased in cases vs controls
Debigare/2008/COPD	10 COPD cases; 6 healthy controls. Age: 70±5 cases; 63± 6 controls. Sex: M. Race/Ethnicity not reported. Matching criteria not reported.	Cachexia: BMI without set threshold. Sarcopenia: **MTCSA by CT below set threshold.**	Vastus lateralis qPCR	*FOXO1* increased in cases vs controls *FOXO3* increased in cases vs controls *IGF-1* increased in cases vs controls *IL-6* NS *TNF* NS
Doucet/ 2007/ COPD	Phase 1: 12 COPD cases; 10 healthy controls. Phase 2: sub-study of 5 COPD cases with preserved muscle mass and 6 COPD cases with low muscle mass. Phase 1: Age: 65±2 cases and controls; Phase 2: Age: 68±3 COPD with preserved muscle mass; 70±1 COPD with low muscle mass; Sex: M Race/Ethnicity not reported. Matching criteria not reported.	Cachexia: Body weight; BMI without set threshold. Sarcopenia: MTCSA by CT without set threshold; FFMI by DXA without set threshold.	QuadricepsqPCR	*FBXO32*: increased in cases vs controls (phase 1); NS in COPD low muscle mass vs COPD preserved muscle mass (phase 2). *TRIM63*: increased in cases vs controls (phase 1); NS in COPD low muscle mass vs COPD preserved muscle mass (phase 2). *FOXO1* increased in cases vs controls (phase 1); NS in COPD low muscle mass vs COPD preserved muscle mass (phase 2). *FOXO3* NS differences in COPD low muscle mass vs COPD preserved muscle mass (phase 2).
Gallagher/2012/ Upper GI Cancer	12 cancer cases; 6 controls (abdominal surgery for non-neoplastic diseases). Age: 65 cases; 58 controls. Sex: M/F 10/2 cases; 4/2 controls. Race/Ethnicity not reported. Matching criteria not reported.	Cachexia: **Weight loss >5% in 6 months**; Body weight; BMI. Sarcopenia: Quadriceps strength (only in cases).	QuadricepsqPCR	*BNIP3* NS *FBXO32* NS *GABARAPL1* NS *TRIM63* NS
Guo/2013/ COPD	Cohort 2: 20 COPD; 10 healthy controls. Age: 68±4 cases; 62±3 controls. Sex M/F: 17/3 cases; 7/3 controls. Race/Ethnicity not reported. Matched by age.	Cachexia: Body weight; BMI without set threshold. Sarcopenia: MTCSA via CT without set threshold.	Vastus lateralis qPCR	*FBXO32* NS *FOXO1* increased in cases vs controls *FOXO3* NS *GABARAPL1* increased in cases vs controls *TRIM63* NS
Kneppers/2017/ COPD	92 COPD cases (further stratified as 53 non-sarcopenic and 39 sarcopenic); 13 healthy controls. Age: 64±7 COPD non-sarcopenic; 66±8 COPD sarcopenic; 64±5 controls. Sex: % Male 60.4% COPD non-sarcopenic; 74.4% COPD sarcopenic; 53.8% controls; Race/Ethnicity not reported. Matching criteria not reported.	Cachexia: BMI Sarcopenia: **ASMI by DXA below set threshold**; FFMI.	Vastus lateralis qPCR	*BECN1* increased in all COPD cases vs controls; NS in COPD sarcopenic vs COPD non-sarcopenic *FBXO32 N*S *FOXO1* increased in COPD sarcopenic vs controls *FOXO3* NS *MAP1LC3B* NS *MSTN* increased in COPD cases vs controls irrespective of sarcopenia; *MYF5* NS *MYOD1* increased in all COPD cases vs controls; *MYOG* increased in COPD cases vs controls irrespective of sarcopenia; *TRIM63* NS
Lemire/2012/ COPD	18 COPD cases; 9 healthy controls. Age: 65±1 cases; 66±3 controls. Sex: M Race/Ethnicity not reported; Matched by age.	Cachexia: BMI without set threshold. Sarcopenia: CSA by CT without set threshold; FFMI by DXA.	Quadriceps qPCR	*FBXO32* increased in cases vs controls *TRIM63* NS
Llovera/1998/ AIDS	3 AIDS cases; 3 healthy controls. Age/Sex/Race/Ethnicity not reported. Matching criteria not reported.	Cachexia: Weight loss >10% from baseline. Sarcopenia: Not assessed.	Deltoid Northern blot	*UBIQUITIN* increased in cases vs controls
Marzetti/2017/ Gastric Cancer	18 gastric cancer cases (further stratified into 9 CC and 9 NCC); 9 controls (abdominal surgery for non-neoplastic diseases). Age: 67±12 CC; 71±9 NCC; 57±16 controls. Sex: F 1 CC; 0 NCC; 1 controls. Race/Ethnicity: Caucasian. Matching criteria not reported.	Cachexia: **Weight loss >5% in 6 months**; BMI. Sarcopenia: Not assessed.	Rectus abdominus qPCR	*TFAM* NS
Murton/2017/ Lung Cancer	4 NSCLC cases; 4 healthy controls. Age: 73±3 cases; 71±2 controls. Sex: M/F 1/3 cases; 2/2 controls. Race/Ethnicity not reported. Matched by age, gender, smoking status, physical activity level.	Cachexia: **Weight loss >5% in 6 months**; BMI Sarcopenia: **lumbar and/or appendicular muscle mass by CT and/or DXA below set threshold.**	Vastus lateralis qPCR	*FBXO32* NS*IL-6* increased in cases vs controls*MSTN* NS*TNF* NS*TRIM63* NS
Op den Kamp/ 2013/ Advanced NSCLC	26 NSCLC cases (further stratified as 10 pre-cachexia and 16 cachexia); 22 healthy controls. Age: 62±10 pre-cachexia; 60± 8 cachexia; 61± 7 controls. Sex: M 80% pre-cachexia; 56% cachexia; 59% controls. Race/Ethnicity not reported. Matching criteria not reported.	Cachexia and pre-cachexia: **according to ref. 37.** Sarcopenia: **ALMI by DXA below set threshold.**	Vastus lateralis qPCR	*BNIP3* increased in cachexia cases vs controls; increased in cachexia cases vs pre-cachexia cases *FBXO32* NS *TRIM63* NS
Op den Kamp/ 2015/NSCLC	26 NSCLC cases (further stratified as 10 pre-cachexia cases; 16 cachexia cases); 22 healthy controls. Age: 61±9 NSCLC; 61±7 controls. Sex: M/F 17/9 NSCLC; 13/9 controls. Race/ Ethnicity not reported. Matched by age and sex.	Cachexia and pre-cachexia: **according to ref. 37.** Sarcopenia: **ALMI by DXA below set threshold.**	Vastus lateralis qPCR	*TFAM* NS
Pessina/2010/ Gastric Cancer	30 gastric cancer; 8 controls (abdominal surgery for benign diseases) Age: 64±3 cases; 64±3 controls. Sex: M/F 17/13 cases; 5/3 controls. Race/Ethnicity not reported. Matched by age.	Cachexia: weight loss without set threshold. Sarcopenia: Not assessed.	Rectus abdominus qPCR	*MYF5* NS *MYOD1* increased in cases vs controls
Plant/2010/ COPD	9 COPD; 9 healthy controls. Age: 64±2 cases; 60±1 controls. Sex: M/F 5/4 cases; 3/6 controls. Race/ Ethnicity not reported. Matched by age.	Cachexia: BMI; % body fat; skinfold thickness; waist circumference. Sarcopenia: CSA by CT without set threshold; quadriceps strength.	Vastus lateralis qPCR	*BECN1* NS *FBXO32* increased in cases vs controls *MAP1LC3B* NS *MSTN* increased in cases vs controls *MYF5* NS *MYOD1* NS *MYOG* NS *TRIM63* NS
Puig-Villanova/ 2015/COPD	41 COPD cases (further stratified into 25 with and 16 without muscle weakness); 19 healthy controls Age: 68±6 cases; 65±8 controls. Sex: M Race/Ethnicity not reported. Matched by age and smoking history.	Cachexia: Weight change and BMI without set thresholds. Sarcopenia: 25% reduction in quadriceps force compared to controls; FFMI.	Vastus lateralis qPCR	*IGF-1* decreased in all COPD cases vs controls; decreased in COPD with muscle weakness vs healthy controls; NS between COPD no muscle weakness and controls *MSTN* decreased in all COPD cases vs controls; decreased in COPD with muscle weakness vs controls; NS between COPD no muscle weakness and controls *MYOD1* NS *MYOG* NS
Ramamoorthy/ 2009/Cancer and AIDS	10 cancer cachexia and 2 AIDS cachexia cases; 4 controls (normal weight, no cancer) Age 56±5 cancer cachexia; 39 and 54 years AIDS cachexia; 59±6 controls. Sex/ Race/Ethnicity not reported. Matching criteria not reported.	Cachexia: % weight loss over 6 months without set threshold. Sarcopenia: Not assessed.	Rectus abdominus or vastus lateralis qPCR	*MYOG* decreased in cases vs controls *TNF* increased in cases vs controls
Remels/2007/ COPD	14 COPD cases (further stratified as 7 COPD cachectic and 7 COPD non- cachectic); 9 healthy controls. Age: 58±13 COPD cachectic; 67±9 COPD non-cachectic; 65±4 controls. Sex: M/F: 4/3 COPD cachectic; 4/3 COPD non-cachectic; 6/3 controls. Race/ Ethnicity not reported. Matched by age.	Cachexia: BMI below set threshold with FFMI below set threshold; BMI Sarcopenia: FFMI	Quadriceps femoris qPCR	*TFAM* NS in all COPD cases vs controls; significantly lower in COPD cachectic cases vs COPD non-cachectic cases.
Stephens/2015/ Upper GI Cancer	92 GI cancer (further stratified as 41 NCC; 51 CC); 15 controls (abdominal surgery for non-malignant/non-inflammatory conditions). Age: 65±10 cases; 56±17 (controls); Sex: M/F 66/26 cases; 8/7 controls. Race/Ethnicity not reported. Matching criteria not reported.	Cachexia: **Weight loss >5% in 6 months**; BMI. Sarcopenia: Not assessed.	Rectus abdominusqPCR	*BNIP3* NS *FBXO32* NS *GABARAPL1* increased in all cancer cases vs controls; NS in cancer cachexia cases vs cancer no cachexia cases *TRIM63* NS
Sun/2012/ Gastric Cancer	102 gastric cancer cases; 29 controls (surgery for benign abdominal diseases). Age: 62± cases; 62±6 controls. Sex: M/F 72/30 cases; 21/8 controls. Race/Ethnicity not reported. Matching criteria not reported	Cachexia: BMI and % weight loss over unspecified length of time without set threshold. Sarcopenia: Not assessed.	Rectus abdominus qPCR	*UBIQUITIN* increased in cases vs controls; higher in cases with >10% weight loss vs cases with <10% weight loss.
Thapaliya/2014/ Alcoholic Cirrhosis	5 alcoholic cirrhosis; 5 controls (donors for liver transplantation or elective abdominal surgery). Age: 49±11 cases; 48±11 controls. Sex: M/F 4/1 cases and controls. Race/ Ethnicity not reported. Matching criteria not reported.	Cachexia: BMI without set threshold. Sarcopenia: Muscle mass of L4, psoas, paraspinal, and abdominal wall by CT without set thresholds.	Rectus abdominus qPCR	*FBXO32* decreased in cases vs controls *TRIM63* decreased in cases vs controls
Vogiatzis/2010/ COPD	10 cachectic COPD (cases); 19 non-cachectic COPD (controls) Age: 63±2 cases; 67±2 controls. Sex: M. Race/Ethnicity not reported. Matched on age and severity of airflow obstruction	Cachexia: Body weight; BMI without set threshold. Sarcopenia: FFMI below set threshold.	Vastus lateralis qPCR	*IGF-1* NS *MSTN* NS *MYOD1* NS *TNF* NS
Yuan/2015/ Cancer	21 cancer cases; 23 controls (surgery for benign abdominal diseases). Age: 59±11 cases; 54±11 controls. Sex: M/F 14/7 cases; 17/6 controls. Race/ Ethnicity not reported. Matching criteria not reported.	Cachexia: BMI without set threshold; Body weight; incidence of weight loss. Sarcopenia: Not assessed.	Rectus abdominus qPCR	*FBXO32* increased in cases vs controls *TRIM63* increased in cases vs controls
Zhang/2013/ CKD	18 CKD cases; 16 healthy controls Age: 67(36–79) cases; 63(46–77) controls; Sex: M/F 11/7 cases; 13/3 controls. Race/Ethnicity not reported. Matched on age and sex.	Cachexia: unintentional weight loss in 3 months without set threshold. Sarcopenia: CSA (area and technique not identified) without set threshold.	Rectus abdominus qPCR	*IL-6* increased in cases vs controls *MSTN* increased in cases vs controls *TNF* increased in cases vs controls

AIDS = acquired immunodeficiency syndrome; ALMI = appendicular lean mass index; ASMI = appendicular skeletal mass index; BIA = bioelectrical impedance; BMI = body mass index; CC = cancer cachexia; CKD = chronic kidney disease; COPD = chronic obstructive pulmonary disease; CSA = cross sectional area; CT scan = computerized tomography scan; DXA = dual x-ray absorptiometry; ESRD = end stage renal disease; FFMI = fat-free mass index; GI = gastrointestinal; HD = hemodialysis; IBW = ideal body weight; M/F = male/female; MTSCA = mid-thigh cross sectional area; NCC = cancer no cachexia; NS = not significant; NSCLC = non-small cell lung cancer; qPCR = quantitative polymerase chain reaction; sqPCR = semi-quantitative polymerase chain reaction; UBW = usual body weight.

^a^ Text in bold indicates the use of standardized definitions of cachexia or sarcopenia whether or not the terms cachexia or sarcopenia are used in the article. Due to the diverse range of assessments used in the articles we analyzed in this systematic review, we include measurement of body weight, BMI and/or weight loss as a proxy for cachexia and assessment of muscle area, muscle mass or muscle strength as a proxy for sarcopenia. However, the terms cachexia and sarcopenia are not used consistently across the 27 articles.

**Table 2:** Genes analyzed in at least three research articles and included in the systematic review.

Gene	Gene Function	Total Number of Articles that Analyzed the Gene	Number of Articles that Found Significant Increase in Cases vs Controls	Number of Articles that Found Significant Decrease in Cases vs Controls	Number of Articles that Found No Significant Difference in Cases vs Controls
*FBXO32* (ATROGIN1)	Protein degradation	12	4 [18, 24, 26, 27]	1 [17]	7 [5, 6, 12–16]
*TRIM63* (MURF1)	Protein degradation	12	2 [18, 24]	1 [17]	9 [5, 6, 12–16, 26, 27]
*UBIQUITIN*	Protein degradation	5	4 [19, 22, 28, 29]	0	1 [30]
*BECN1*	Autophagy	3	1^a^ [6]	0	2 [27, 31]
*GABARAPL1*	Autophagy	3	2^b^ [5, 15]	0	1 [12]
*MAP1LC3B2* (LC3B)	Autophagy	3	1^c^ [31]	0	2 [6, 27]
*SQSTM1 (p62)*	Autophagy	3	1 [15]	0	2 [6, 31]
*BNIP3*	Apoptosis	4	1^d^ [16]	0	3^e^ [5, 12, 31]
*FOXO3*	Apoptosis	4	1 [32]	0	3^f^ [6, 15, 18]
*MSTN*	Muscle differentiation/ growth (Negative regulator)	7	3 [6, 25, 27]	2^g^ [14, 33]	2 [13, 23]
*MYOD1*	Muscle differentiation/ growth	5	2 [6, 34]	0	3 [23, 27, 33]
*MYOG*	Muscle differentiation/growth	4	1 [6]	1 [35]	2 [27, 33]
*MYF5*	Muscle differentiation/growth	3	0	0	3 [6, 27, 34]
*FOXO1*	Insulin/IGF1 pathway	4	4^h^^,^^i^ [6, 15, 18, 32]	0	0
*IGF1*	Insulin/IGF1 pathway	4	1 [32]	2^j^ [14, 33]	1 [23]
*TNF*	Inflammation	5	2 [25, 35]	0	3 [13, 23, 32]
*IL-6*	Inflammation	3	2 [13, 25]	0	1 [32]
*TFAM*	Mitochondrial transcription regulation	3	0	0	3^j^ [20, 21, 36]

Gene function was assigned according to www.genecards.org Some genes, particularly FOXO1 and FOXO3, can be classified as part of multiple pathways.

^a^ Kneppers 2017 found significant increase in all chronic obstructive pulmonary disease (COPD) cases vs controls but no significant differences between COPD sarcopenia and COPD no sarcopenia

^b^ Stephens 2015 found significant increase in all cancer cases vs controls but no significant differences in cancer cachexia vs cancer no cachexia

^c^ Aversa 2016 found a significant increase in cancer cachexia cases vs controls

^d^ Op den Kamp 2013 found significant increase in NSCLC cachexia vs controls; increased in NSCLC cachexia vs precachexia cases

^e^ Stephens 2015 found trend towards increase in all cancer cases vs controls (p = 0.058)

^f^ Doucet 2017 found no significant differences in COPD low muscle mass vs COPD preserved muscle mass

^g^ Puig-Villanova 2015 found significant decrease in all COPD cases vs healthy controls; decreased in COPD with muscle weakness vs healthy controls; NS differences between COPD no muscle weakness and healthy controls

^h^ Doucet 2007 found significant increase in all COPD cases vs controls but no significant differences between COPD low muscle mass and COPD preserved muscle mass

^i^ Kneppers 2017 found significant increase in COPD sarcopenic vs healthy controls

^j^ Remels 2007 found no significant difference between all COPD cases and controls but significantly lower in COPD cachectic vs COPD non-cachectic

### Development of the data extraction tool

We developed a data extraction tool (S2 File) to collect relevant data from the articles and assess the quality of the data. We used the Strengthening the Reporting of Observational Studies in Epidemiology (STROBE) [37] criteria as a guide in determining which information to include.

One of the goals of this systematic review was to critically evaluate the quality of the study design and strength of the evidence, including criteria for assessing cachexia and/or sarcopenia. A standard definition of cachexia was developed via consensus in 2008; it includes a 5% weight loss in ≤12 months or a body mass index (BMI) <20 kg/m^2^ plus three out of five criteria: decreased muscle strength, fatigue, anorexia, low fat-free mass index, and abnormal biochemistry, including increased inflammatory markers, anemia and decreased albumin [1]. Specific criteria for cancer cachexia were developed in 2011; they include a 5% weight loss in ≤6 months (in the absence of simple starvation), or BMI < 20kg/m^2^ with any degree of weight loss >2%, or appendicular skeletal mass index (ASMI) consistent with sarcopenia (<7.26 kg/m^2^ for men and <5.45 kg/m^2^ for women) with any degree of weight loss >2% [38]. Sarcopenia is defined as two standard deviations below the mean reference value for healthy young adults depending on the measurement technique [4, 39]. Sarcopenia can be measured using DXA, computerized tomography (CT), magnetic resonance imaging (MRI), bioimpedance analysis (BIA), as well as total or partial body potassium or anthropometry. The gold standards for assessing muscle mass are the CT and MRI, with DXA being the preferred alternative method [4]. Due to the diverse range of assessments used in the articles we analyzed in this systematic review, we included measurement of body weight, BMI and/or weight loss as a proxy for cachexia, as well as assessment of muscle area, muscle mass or muscle strength as a proxy for sarcopenia. It must be noted that the terms cachexia and sarcopenia are not used consistently across the 27 articles included in this review and are not all consistent with the standard definitions, particularly for those articles published prior to the development of the consensus definitions.

We also assessed articles based on the strength of their molecular analysis. Quantitative Polymerase Chain Reaction (qPCR) is a technique used to accurately and reliably assess gene expression [40]. Minimum Information for Publication of q-PCR Experiments (MIQE) Guidelines were developed to evaluate the reliability of results, promote consistency among laboratories and ensure transparency for replicating experiments when using qPCR [41]. The MIQE guidelines include documentation of the reference gene and the primer sequence used and repeatability or replication of sample results [41]. In addition, laboratory research and clinical trials should ensure that whoever sets up the experiment does not measure the outcome [42] or should be blinded as to the identity of cases and controls to avoid bias when measuring the outcome. We assessed the quality of the molecular analysis in the studies included in this systematic review by use of validated reference genes, experimental replicates, reporting of primer/probe sequence and blinded analysis of gene expression.

We also assessed the study’s statistical power [41] or whether sample size was determined based on the outcome of gene expression or other outcome and adequacy of the statistical methods. Moreover, we determined whether the study design included matching criteria for cases and controls and whether the population included in the study was considered representative of the population at large affected by the specific condition.

### Data extraction

Three researchers (GF, CB, AM) reviewed eligible articles and extracted relevant data via the data extraction tool (S2 File). To reduce selection bias and minimize error, each article was reviewed by two researchers (GF and CB; GF and AM) and discrepancies were discussed and resolved via consensus. Each article was also assessed for quality by two researchers and discrepancies were resolved via consensus.

## Results

The 27 articles that investigated the 18 genes are summarized in Table 1. Table 2 provides an overall summary of the 18 genes by direction of differential gene expression (significant increase, significant decrease or no significant difference between cases and controls) to determine patterns in gene expression overall and across gene function, as determined using www.genecards.org.

### Subject characteristics and quality of the study design

Data from the 27 articles were compared and tabulated but not meta-analyzed. Table 3 provides a summary of subject characteristics for the 27 studies included in the systematic review. Participants were approximately 60 years of age and majority male with race/ethnicity only reported in one study. Sample sizes were highly variable and none of the studies mentioned whether their subjects were representative of the population affected by the specific disease.

**Table 3:** Subject characteristics for the 27 studies included in the systematic review.

Subject Characteristics	Cases	Controls
Mean Age (years)	60.5 (range of 43–71)	58.6 (range of 42–65)
Sex (% male)	70.5	69.7
Race/Ethnicity	Only 1 study reported race/ethnicity as Caucasian	
Sample Size (mean ± standard deviation)	25.6 ± 26.6	11.7 ± 6.8
Representative population	None of the studies mentioned this criterion	

Table 4 provides a summary of study design characteristics for the 27 articles included in the systematic review. The majority of studies investigated cachexia and/or sarcopenia in COPD or cancer, with variability amongst the studies in the choice of control group. Eleven articles included a sub-analysis of cases based on either presence/absence or severity of cachexia and/or sarcopenia. Most studies did not report matching criteria for cases and controls; the few that reported this criterion primarily matched on age alone. The articles also differed in the location of muscle biopsied, with the majority using samples from rectus abdominus or quadriceps. The vast majority assessed gene expression by PCR, while a few studies published before 2004 used Northern Blot.

**Table 4:** Summary of the study design characteristics for the 27 studies included in the systematic review.

Study Design Characteristics	Type	# Articles
Diseases Studied	Cancer	14
	COPD	9
	CKD	2
	HIV/AIDS	1
	Cirrhosis	1
Controls Used/Sub-analysis	Healthy controls	17
	Surgical procedures for benign disease	9
	Chronic disease but no cachexia/ sarcopenia	1
	Sub-analysis*	11
Matching Criteria	Age alone	7
	Age and sex	3
	Age and airflow obstruction	1
	Not reported	16
Assessment of Cachexia/ Sarcopenia**	Cachexia	15
	Sarcopenia	9
	Cachexia and Sarcopenia	3
Muscles Biopsied	Rectus abdominus	13
	Quadriceps	13 (9 vastus lateralis)
	Deltoid	1
Technique used for Molecular Analysis	qPCR	23
	Northern blot	4

COPD = chronic obstructive pulmonary disease, CKD = chronic kidney disease, HIV/AIDS = human immunodeficiency virus/ acquired immunodeficiency syndrome, qPCR = quantitative polymerase chain reaction.

*Articles that included a sub-analysis of cases based on presence or absence of cachexia and/or sarcopenia or degree of weight loss. See Table 1 for details.

**Under the term cachexia we include assessment of weight loss and or body size. Under the term sarcopenia we include assessment of muscle mass or muscle strength. However, the terms cachexia and sarcopenia are not used consistently throughout the 27 articles.

More than half of the articles investigating cachexia defined it as at least 5% weight loss or BMI <20 kg/m^2^, which is consistent with standard definitions though it does not include all the criteria that are part of the standard definitions [1, 38]. One-third of the articles investigated sarcopenia as quantified by DXA, CT scan or LMI. While many articles assessed criteria that can be construed as part of the assessment of both cachexia and sarcopenia, only three articles included measures of both cachexia and sarcopenia that conform to standard definitions.

As detailed in Table 1, the articles differed in their assessment of cachexia and sarcopenia. There was variability both in the time frame used to define weight loss (i.e., <6 months, which is consistent with cancer cachexia criteria; <12 months, which is consistent with cachexia criteria; weight loss from usual body weight) and in the amount of weight loss (5% vs 10%). Two articles defined cachexia using BMI or BMI in conjunction with fat-free mass index (FFMI). Furthermore, various criteria were used to assess and/or define sarcopenia, with the majority using statistical differences in cross sectional area (CSA) or FFMI/LMI between cases and controls but no reference to set standard thresholds. Only five articles assessed sarcopenia using criteria consistent with the standardized definitions and using set reference thresholds rather than differences between cases and controls specific to their own study sample.

### Quality of molecular analysis

Of the 27 studies included in the systematic review, 23 used qPCR for evaluating gene expression whereas four used Northern blot. None of the studies fulfilled all four criteria used by the researchers for assessing the quality of the molecular analysis (Table 5). Although the majority of studies reported primer/probe sequences, most studies did not indicate the method for validation of the reference gene(s) used or the number of replicates performed. Only one of the 27 articles powered on the outcome of gene expression [23] and one other article powered on a different outcome [33] whereas the remaining 25 articles did not mention statistical power at all. Finally, none of the studies reported on blinding strategies.

**Table 5:** Number of articles that reported (Yes) or did not report (No) on five standards for evaluating the molecular analysis used for measuring gene expression in skeletal muscle of individuals with cachexia and/or sarcopenia and their controls.

The Article Reported the Following Standards:	Yes	No
Primer/ Probe Sequence	25	2
Sample Replicates	8	19
Validation of reference gene	4	23
Powered on Gene Expression	1	26^a^
Blinded Analysis	0	27

^a^ Puig-Villanova, 2015 powered on quadriceps muscle velocity contraction but did not power on the outcome of gene expression

### Differential gene expression

As shown in Table 2, *FBXO32 (ATROGIN1*) and *TRIM63 (MURF1)* were the two most studied genes, collectively analyzed in 12 out of 27 articles. However, findings were not consistent, with the majority of articles reporting no statistically significant differences between cases and controls for expression of either *FBXO32* (n = 7) or *TRIM63* (n = 9).

We sought to determine whether the disease studied in the original articles dictated the direction of change for *FBXO32* and *TRIM63* (Table 6). Of the five studies that evaluated expression of *FBXO32* in COPD, three reported a significant increase in cases versus controls while two reported no significant differences between groups. The same five studies also investigated expression of *TRIM63*, with one observing a significant increase in cases versus controls and four finding no significant differences between the two groups. Six studies evaluated expression of both *FBXO32* and *TRIM63* in patients with cancer. Of these, one study found a significant increase in the expression of both genes in cases versus controls while the remaining five studies reported no significant differences between the two groups. The only article that found a significant decrease in both genes in cases versus controls studied individuals with alcoholic cirrhosis. Because no clear patterns of gene expression emerged based on disease, we conclude that the type of disease does not dictate direction of change for *FBXO32* or *TRIM63* in the context of cachexia/sarcopenia. We also assessed whether the type of controls used in the study might affect the results. Each of the 12 studies that evaluated *FBXO32* or *TRIM63* included a healthy control group as the main comparator. However, two of these 12 studies performed a sub-analysis, comparing cases with sarcopenia to cases without sarcopenia [6, 18]. Both studies found no significant differences for expression of either *FBXO32* or *TRIM63* in cases with sarcopenia versus cases without sarcopenia.

**Table 6:** Results for FBXO32 (ATROGIN1) and TRIM63 (MURF1) by disease/condition studied in the original research articles.

Condition Studied	Significant Increase in cases vs controls		Significant Decrease in cases vs controls		No Significant Difference between cases and controls*	
	*FBXO32*	*TRIM63*	*FBXO32*	*TRIM63*	*FBXO32*	*TRIM63*
COPD	3 [18, 26, 27]	1 [18]			2 [6, 15]	4 [6, 15, 26, 27]
Cancer	1 [24]	1 [24]			5 [5, 12–14, 16]	5 [5, 12–14, 16]
Alcoholic cirrhosis			1 [17]	1 [17]		

COPD = chronic obstructive pulmonary disease

*Refs. 6 and 18 performed a sub-analysis comparing cases with sarcopenia to cases without sarcopenia. Neither study found a significant difference for either FBXO32 or TRIM63.

*FOXO1* was the only gene consistently significantly increased in cases versus controls in all studies that investigated its expression (n = 4). All four studies that investigated *FOXO1* did so in participants with COPD. Two of the studies [6, 18] not only compared cases with COPD versus healthy controls, finding significantly higher levels in cases, but also compared a group of COPD patients with sarcopenia to a group of COPD patients without sarcopenia. Neither study found a significant difference in expression of *FOXO1* between COPD patients with sarcopenia and COPD patients without sarcopenia.

Four of the five articles measuring *UBIQUITIN* found a statistically significant increase in cases versus controls. Of these, three articles investigated gene expression in individuals with gastric cancer [22, 28, 29] and one in acquired immunodeficiency syndrome (AIDS) [19]. The fifth article investigated *UBIQUITIN* expression in CKD (30) and found no significant difference between cases and controls. Sub-analysis by weight loss, performed in two of the five studies, led to contradictory findings. One study demonstrated higher *UBIQUITIN* in patients with >10% weight loss compared to those with <10% weight loss [22] whereas another study found expression of *UBIQUITIN* in cases did not correlate with degree of weight loss [28].

Data analysis revealed contradictory findings for three genes (*MSTN*, *MYOG*, *IGF1)* but the type of disease did not explain these contradictions. For instance, the three studies that found a significant increase in *MSTN* expression in cases versus controls investigated COPD and CKD, the two studies that found a significant decrease in cases versus controls investigated COPD and gastric cancer, whereas the two studies that found a nonsignificant difference in *MSTN* expression in cases versus controls investigated COPD and lung cancer. Similar observations were made for *MYOG* and *IGF1*. Therefore, no clear patterns of gene expression emerged for these three genes based on disease. Sub-analysis by type of control group also revealed contradicting findings for this group of genes. For *MSTN*, some studies found a differential expression in cases versus healthy controls irrespective of the presence of sarcopenia or weight loss [6, 14] whereas others indicated decreased expression only for those cases in which sarcopenia was present [33]. Of the two studies that found no significant differences in *MSTN* expression in cases versus controls, one used a healthy control group [13] while the other used a group of patients with the same condition but without sarcopenia [23]. A comparable pattern was observed for *IGF1*.

The remaining 11 genes fell into three categories: 1) those with no articles finding a significant decrease in gene expression in cases versus controls (*BECN1*, *GABARAPL1*, *MAP1LC3B2* (LC3B), *SQSTM1 (p62)*, *BNIP3*, *FOXO3*, *MYOD1*, *TNF*, *IL-6*); 2) those with no articles finding a significant increase in gene expression in cases versus controls (*TFAM*); and 3) those with articles finding no significant differences in gene expression in cases versus controls (*MYF5*).

### Gene functions

To determine if any pattern of gene expression might emerge, we grouped the 18 genes studied in at least three articles by main function according to www.genecards.org (Table 2). For the function of protein degradation, 59% of studies found no statistical difference in gene expression between cases and controls, 34% found significantly higher levels in cases versus controls while 2% found significantly lower levels in cases versus controls. Results for genes grouped under the functions of autophagy and inflammation were approximately equally split between those showing significantly higher levels in cases versus controls and those showing no significant differences; no study indicated the presence of significantly lower levels in cases versus controls for genes grouped under these two functions. Similarly, no study found a significant decrease in gene expression in cases versus controls for the function of apoptosis, but here the majority of results (75%) indicated no significant differences between cases and controls. For the function of muscle differentiation/growth, 53% of studies indicated lack of significant differences between groups, 31% indicated significantly higher gene expression in cases versus controls, while 16% indicated significantly lower expression in cases versus controls. The insulin/IGF1 pathway included 62% of studies showing an increase in cases versus controls, 25% showing a significant decrease and 12.5% (one study) showing no significant difference. Finally, none of the three studies grouped under the function of mitochondrial transcription found any statistical difference in gene expression in cases versus controls.

## Discussion

The purpose of this systematic review was to summarize and assess the strength of the current evidence on gene expression in skeletal muscle of individuals with chronic disease-associated cachexia and/or sarcopenia (cases) compared to controls. We found that studies recruited a majority of male subjects, approximately 60 years of age, which may be due to the prevalence of these chronic conditions in the older male population. Sample size was highly variable among studies, with the majority (85%) including </ = 30 cases, which is comparable to findings reported in a recent state of the science review of muscle biopsies in surgical cancer patients [43]. Obtaining muscle biopsy samples from human subjects can be challenging, particularly in frail individuals who may have several contraindications to the procedure [44], a factor that may have contributed to the limited sample size of some of the studies included in this review. Remarkably, only two reports [23, 33] indicated how the study was powered, with only one powering on the outcome of gene expression [23]. More than 92% of the studies did not report how the sample sizes were determined, which is comparable to results found for muscle biopsies in surgical cancer patients, in which 96% of the studies did not justify sample size [43]. Without disclosure of how a study is powered, it is not known if the sample size is sufficient to detect a true difference in gene expression in cases versus controls. Thus, for the almost totality of studies evaluated in this review, lack of statistical significance cannot be construed as no difference between cases and controls.

The STROBE guidelines [37] indicate the importance of reporting descriptive data for study participants. However, not all studies included descriptive data, which makes reliable comparison of results across studies challenging, in agreement with conclusions of the state of the field performed by other investigators [43]. For instance, almost 20% of the studies did not report the sex of the participants. Of those that did report sex, 70% of the cases and controls were male, which limits the generalizability of the gene expression data. Only one study reported the race/ethnicity of its participants. This is important, as lean body mass and risk for disease may differ across racial and ethnic groups as does BMI [45], and this difference may have varying implications in the association between degree of sarcopenia and muscle gene expression. Moreover, lack of diversity may be a sign that the study sample is not representative of the general population affected by the specific disease, an issue that went unaddressed in the studies we examined. Anoveros-Barrera et al [43] found a similar lack of representativeness in the cancer studies included in their literature review.

The articles included in this systematic review were not homogenous in terms of study design, including the choice of control group, matching criteria, assessment of cachexia and/or sarcopenia and muscles biopsied. Control groups ranged from healthy individuals to patients undergoing various surgical procedures, to individuals with the same disease as the cases but no cachexia/sarcopenia. The use of diverse comparison groups makes it difficult to assess differences in gene expression between cases and controls. Establishing standards on which group is best to use for comparison of gene expression studies would remedy this issue.

Less than half of the studies reported matching criteria for cases and controls. Of these criteria, age was the most common and sex came second, with only 11% (n = 3) of the studies matching on both age and sex. Considering the sexual dimorpism that exists in skeletal muscle anabolism and catabolism pathways [43], it is essential that gene expression studies include a representative sample of both sexes and cases and controls are matched on sex. Remarkably, important variables such as race/ethnicity were not included as matching criteria in any of the studies while smoking status was only considered in a small subgroup of reports. If cases and controls are not appropriately matched, then confounders may impact the outcome of gene expression.

Importantly, the studies differed in how they defined and assessed cachexia and sarcopenia. Though this is inevitable for studies performed before the establishment of initial consensus definitions (2008 for cachexia, 2010 for sarcopenia, 2011 for cancer cachexia) [1, 4, 38], the problem persists in reports published several years after the establishment of standardized criteria. These discrepancies in how cachexia and sarcopenia are defined and measured, as well as the diverse, interchangeable terminology adopted in the various studies, with the added confounding factor of overlapping characteristics between sarcopenia and cachexia, all contribute to the heterogeneity of the results and make it difficult to compare findings across studies and/or diseases. The reports also differed in the muscles biopsied for analysis of gene expression, which may represent an additional source of variability.

We assessed the quality of molecular analysis using the MIQE guidelines [41], which include use of validated reference genes, number of replicates, reporting of primer/probe sequences and blinded analysis of gene expression. None of the studies fulfilled all four criteria. While 93% of studies reported the primer/probe sequence used, only 15% mentioned the process used to validate reference genes, if any such process was used. This is important, as genes whose expression is not affected by disease status or confounding variables should be selected as housekeeping references. Without disclosure of the validation process for the reference gene(s), it is difficult to assess whether the researchers truly measured change in expression levels of the genes under investigation. Moreover, only 30% of the studies assessed their samples at least in duplicate, an important step to ensure no bias or error occurred when assessing gene expression and for ensuring the results are accurate. It is also important to note that no study reported or mentioned blinding strategies employed during the process of molecular analysis, which is integral for eliminating bias. Finally, only one study [23] powered sample size on the outcome of gene expression, whereas one other study [33] powered on a different outcome. None of the other studies (>92% of the whole sample) reported or mentioned statistical power at all. The issue of statistical power is critical, as an appropriate sample size is needed to detect true statistical differences between cases and controls. Due to this issue, lack of statistical significance in the reports we analyzed cannot be construed as no difference between cases and controls as the studies may not have been powered for the specific outcome.

Despite the heterogeneity of the studies, we found a few patterns in gene expression. *FOXO1* was the only gene for which all studies (n = 4) found a statistically significant increase in cases versus controls. These four studies investigated *FOXO1* mRNA expression in cases with COPD versus healthy controls. This finding is consistent with results from animal models [10] and cancer patients [46] and indicates that *FOXO1* may be involved in skeletal muscle wasting [18]. However, the two studies that perfomed a sub-analysis did not observe any significant differences in *FOXO1* expression in COPD cases with sarcopenia versus COPD cases without sarcopenia. Due to lack of reporting about statistical power, we are unable to determine whether this result is to be attributed to pathophysiological factors or to study design issues. In addition, *UBIQUITIN*, which is involved in protein degradation, was statistically increased in cases versus controls in four out of five studies. This finding suggests an upregulation of the ATP-ubiquitin-dependent proteolysis pathway in skeletal muscle of cancer and AIDS patients [29].

The current understanding of the pathophysiology of cachexia-associated sarcopenia led us to expect increased markers of apoptosis, autophagy, and inflammation in individuals with cachexia/sarcopenia versus controls. However, the majority of studies found no significant differences in expression of genes involved in these functions in cases versus controls, perhaps as a consequence of lack of statistical power.

Results from gene expression studies in animal models do not always translate to humans, which may be due to the inability to replicate the complex mechanisms and disease-specific factors that impact human skeletal muscle [2]. For example, animal models of muscle atrophy demonstrated a consistent increase in expression of *FBXO32* and *TRIM63* [8–11] but the same results have not been consistently confirmed in human studies [5, 6, 12–17]. The majority of studies included in this systematic review that investigated *FBXO32* and *TRIM63* found no statistical difference in gene expression between cases and controls, which may again be due to lack of statistical power.

Lack of translatability from animal models to humans may also be attributed to the small number of studies evaluating the same genes in animals and humans. In this systematic review, we identified 151 genes studied in 34 articles. However, the majority of genes (n = 133, S3 File) were only studied in one or two articles, which did not provide enough evidence to summarize patterns of gene expression. Only 18 genes were investigated in at least three articles. Concerted efforts should be made to focus on the expression of a critical number of genes in human cachectic/sarcopenic skeletal muscle to identify similarities or dissimilarities with animal models.

While the current systematic review is the first to examine gene expression in human skeletal muscle of individuals with cachexia and/or sarcopenia associated with a range of chronic diseases, there are several limitations to report. First, the researchers included both cachexia and sarcopenia in the systematic search used to identify potential studies for inclusion despite different criteria used to define these conditions. This inclusion of both conditions may have contributed to the heterogeneity of the results. Secondly, the researchers summarized the current evidence on gene expression but did not meta analyze the raw data, which may have identified additional patterns in gene expression in cases versus controls. Thirdly, the data on gene expression included in this systematic review came primarily from cross-sectional studies that evaluated skeletal muscle at one point in time and not longitudinally; therefore, no conclusions can be drawn about causation of gene expression or changes in gene expression over time in chronic disease-associated cachexia and/or sarcopenia.

## Conclusion

In conclusion, our systematic review on gene expression in the skeletal muscle of humans with chronic disease-associated cachexia and/or sarcopenia (cases) compared to controls found that the evidence may not be powered appropriately and is not homogenous; therefore, it is difficult to compare gene expression results across studies and diseases. Standards should be developed for study design, including determination of appropriate sample sizes, consistency in the use of control groups, using standardized definitions for cachexia and/or sarcopenia and appropriate methods of measurement, following MIQE guidelines to promote transparency and ensure reliability of molecular analysis results, and focusing research efforts on a critical number of genes in human muscle.

## Supporting information

S1 File Search strategy. Search Strategy Used for the Systematic Review on Gene Expression in Skeletal Muscle of Individuals with Chronic-Disease Associated Cachexia or Sarcopenia.
(DOCX)

S2 File Data extraction tool. Data Extraction Tool Developed and Used by the Researchers to Extract Data from the Original Research Articles.
(DOCX)

S3 File List of 133 genes analyzed in less than three research articles. (DOCX)

S4 File PRISMA 2009 checklist. (DOC)

S5 File PRISMA 2009 flow diagram. (DOC)
