# Supplementary material for: Expression of genes in the skeletal muscle of individuals with cachexia/sarcopenia: A systematic review
Source: PLoS One. 2019 Sep 9;14(9):e0222345. doi: 10.1371/journal.pone.0222345 (PMC6733509; doi:10.1371/journal.pone.0222345)
Supplement: S1 File — Search Strategy Used for the Systematic Review on Gene Expression in Skeletal Muscle of Individuals with Chronic-Disease Associated Cachexia or Sarcopenia. (DOCX) [file pone.0222345.s001.docx]

**S1 File. Search Strategy.**  Search Strategy Used for the Systematic Review on Gene Expression in Skeletal Muscle of Individuals with Chronic-Disease Associated Cachexia or Sarcopenia

**PubMed**

#1 "Cachexia"[mesh]

#2 "Atrophy"[mesh]

#3 "Weight Loss"[mesh]

#4 "Wasting Syndrome"[Mesh]

#5 "wasting"[tw]

#6 "weight loss"[tw]

#7 "sarcopenia"[tw]

#8 "cachexia"[tw]

#9 "emaciated"[tw]

#10 "emaciation"[tw]

#11 "precachexia"[tw]

#12 "malnutrition"[tw]

#13 "cachexic"[tw]

#14 "Cachectic"[tw]

#15 #1 OR #2 OR #3 OR #4 OR #5 OR #6 OR #7 OR #8 OR #9 OR #10 OR #11 OR #12 OR #13 OR #14

#16 "Proteins"[mesh]

#17 "RNA, Messenger"[mesh]

#18 "Gene Expression"[mesh]

#19 "protein"[tw]

#20 "mRNA"[tw]

#21 "western blotting "[tw]

#22 "western blot"[tw]

#23 "transcriptional regulation"[tw]

#24 "polymerase chain reaction"[tw]

#25 "PCR"[tw]

#26 "proteins"[tw]

#27 "gene expressions"[tw]

#28 "gene expression"[tw]

#29 "western blots"[tw]

#30 "polymerase chain reactions"[tw]

#31 "messenger RNAs"[tw]

#32 "messenger RNA"[tw]

#33 "mRNAs"[tw]

#34 #16 OR #17 OR #18 OR #19 OR #20 OR #21 OR #22 OR #23 OR #24 OR #25 OR #26 OR #27 OR #28 OR #29 OR #30 OR #31 OR #32 OR #33

#35 "Muscles"[mesh]

#36 "muscle"[tw]

#37 "muscles"[tw]

#38 "myogenic"[tw]

#39 "muscular"[tw]

#40 "intramuscular"[tw]

#41 #35 OR #36 OR #37 OR #38 OR #39 OR #40

#42 #15 AND #34 AND #41

#43 #42 NOT (animals[mesh] NOT humans[mesh]))

#44 #43 NOT "review"[Publication Type])

**EMBASE**

#1 'cachexia'/exp

#2 atrophy'/exp

#3 'body weight loss'/exp

#4 'wasting syndrome'/exp

#5 'wasting':ti,ab,kw

#6 'weight loss':ti,ab,kw

#7 'sarcopenia':ti,ab,kw

#8 'cachexia':ti,ab,kw

#9 emaciat*':ti,ab,kw

#10 'malnutrition':ti,ab,kw

#11 'precachexia':ti,ab,kw

#12 'cachexic':ti,ab,kw

#13 'Cachectic':ti,ab,kw

#14 #1 OR #2 OR #3 OR #4 OR #5 OR #6 OR #7 OR #8 OR #9 OR #10 OR #11 OR #12 OR #13

#15 'protein'/exp

#16 'messenger RNA'/exp

#17 'gene expression'/exp

#18 'protein*':ti,ab,kw

#19 'mRNA*':ti,ab,kw

#20 'western blot*':ti,ab,kw

#21 'transcriptional regulation*':ti,ab,kw

#22 'polymerase chain reaction*':ti,ab,kw

#23 'PCR*':ti,ab,kw

#24 'gene expression*':ti,ab,kw

#25 'messenger RNA*':ti,ab,kw

#26 #15 OR #16 OR #17 OR #18 OR #19 OR #20 OR #21 OR #22 OR #23 OR #24 OR #25

#27 'muscle'/exp

#28 'muscle*':ti,ab,kw

#29 'myogenic':ti,ab,kw

#30 'muscular':ti,ab,kw

#31 'intramuscular':ti,ab,kw

#32 #27 OR #28 OR #29 OR #30 OR #31

#33 #14 AND #26 AND #32

#34 #33NOT ([animals]/lim NOT [humans]/lim)

#35 #34 NOT ('review'/it OR 'conference abstract'/it OR 'conference paper'/it)

**CINAHL**

#1 (MH "Weight Loss+")

#2 (MH "Atrophy+")

#3 (MH "Cachexia")

#4 (MH "Wasting Syndrome+")

#5 AB("wasting") OR TI("wasting")

#6 AB("weight loss") OR TI("weight loss")

#7 AB("sarcopenia") OR TI("sarcopenia")

#8 AB("cachexia") OR TI("cachexia")

#9 AB("emaciat*") OR TI("emaciat*")

#10 AB("malnutrition") OR TI("malnutrition")

#11 AB("precachexia") OR TI("precachexia")

#12 AB("Cachectic") OR TI("Cachectic")

#13 AB("cachexic") OR TI("cachexic")

#14 S1 OR S2 OR S3 OR S4 OR S5 OR S6 OR S7 OR S8 OR S9 OR S10 OR S11 OR S12 OR S12 OR S13

#15 (MH "gene expression+")

#16 (MH "Proteins+")

#17 (MH "Gene Expression Profiling+")

#18 AB("protein*") OR TI("protein*")

#19 AB("mRNA*") OR TI("mRNA*")

#20 AB("western blot*") OR TI("western blot*")

#21 AB("transcriptional regulation*") OR TI("transcriptional regulation*")

#22 AB("polymerase chain reaction*") OR TI("polymerase chain reaction*")

#23 AB("PCR*") OR TI("PCR*")

#24 AB("gene expression*") OR TI("gene expression*")

#25 AB("messenger RNA*") OR TI("messenger RNA*")

#26 S15 OR S16 OR S17 OR S18 OR S19 OR S20 OR S21 OR S22 OR S23 OR S24 OR S25

#27 (MH "Muscles+")

#28 AB("muscle*") OR TI("muscle*")

#29 AB("myogenic") OR TI("myogenic")

#30 AB("muscular") OR TI("muscular")

#31 AB("intramuscular") OR TI("intramuscular")

#32 S27 OR S28 OR S29 OR S30 OR S31

#33 S32 AND S26 AND S14

#34 S33 NOT (MH "Animals" NOT MH "Human)
