# Supplementary material for: Expression of genes in the skeletal muscle of individuals with cachexia/sarcopenia: A systematic review
Source: PLoS One. 2019 Sep 9;14(9):e0222345. doi: 10.1371/journal.pone.0222345 (PMC6733509; doi:10.1371/journal.pone.0222345)
Supplement: S2 File — Data Extraction Tool Developed and Used by the Researchers to Extract Data from the Original Research Articles. (DOCX) [file pone.0222345.s002.docx]

**S2 File. Data Extraction Tool.** Data Extraction Tool Developed and Used by the Researchers to Extract Data from the Original Research Articles

**Gene Expression in Skeletal Muscle of Individuals with Cachexia and/or Sarcopenia: Data Extraction**

**Citation**:

**Is the article relevant to the key question for the systematic review**? Analyze using PICO

Population 1- Patients with cachexia/sarcopenia/muscle atrophy/wasting **YES or** **NO**

OR

Population 2- diseases associated with cachexia/ sarcopenia/ muscle atrophy/ wasting (COPD, CHF, cancer, HIV, RA, hepatitis);

**YES or** **NO; if yes, save these articles for separate analysis**

Intervention- muscle biopsy; **YES or** **NO**

Comparison- Healthy subjects or patients with same diagnosis but without cachexia/sarcopenia/muscle atrophy/wasting;

**YES or** **NO**

Outcome- gene expression in skeletal muscle; **YES or** **NO**

**If no, reason for rejection**: Paper not relevant to key question; other reason:

Description of the Study:

Study Design:

**Cross-sectional study- looking at prevalence of disease and/or exposure at a specific point in time**

**Cohort study- group of individuals are exposed to a factor of interest then followed over time**

**Case-control study- determine if an exposure is associated with an outcome (e.g., disease, condition); look retrospectively to determine if exposure is associated with cases or controls**

**Longitudinal (prospective) study- repeated measures over time**

**Randomized control trial-a treatment or intervention is conducted to determine cause and effect between the treatment/ intervention and a particular outcome**

Purpose of the study:

Participants (n): cases:

Sex:

Age:

Race/ ethnicity:

Disease diagnosis and stage:

Location of muscle biopsy:

List the genes measured: document in Genes Excel Spreadsheet

Main findings of gene expression: document in Genes Excel spreadsheet

Technique used for analyzing gene expression:

Other:

**Data extraction completed by**:

| **Study Design** | | **Points** | **Comments** |
| --- | --- | --- | --- |
| Is sarcopenia present as assessed by lean mass? | Yes (1) No (0)  Not reported/ not addressed (NA) |  | If lean mass is assessed, which method was used?  DXA  MRI  CT scan  Measurement of lean mass index  Other: _______ |
| Is cachexia present as assessed by weight loss of at least 5% in 12 months or less or BMI <20 kg/m2? | Yes (1)  No (0)  Not reported/ not addressed (NA) |  | If weight loss is consistent with the definition of cachexia, which criteria was used?  at least 5 % weight loss in 12 months or less  BMI <20 kg/m2 |
| Cases and controls are matched  (e.g., sex, age, race/ethnicity, smoking status, activity levels) | Adequately addressed (2)  Poorly addressed (1)  Not addressed/ not reported (0) |  |  |

| **Molecular Analysis** | | **Points** | **Comments** |
| --- | --- | --- | --- |
| Is the quality of the molecular analysis appropriate?  Assessment of appropriateness includes:  validation of the reference gene;  documentation of primer/probe sequence;  sample replicates;  blinded analysis | Adequately addressed (2)  Poorly addressed (1)  Not addressed/ not reported (0) |  |  |

| **Statistics** |  | **Points** | **Comments** |
| --- | --- | --- | --- |
| The study was powered on the following variable(s): | Gene expression (2)  different outcome (1)  Not reported/ not addressed (0) |  |  |
| Are statistical methods adequately addressed? | Yes (1)  No (0) |  |  |
| If missing data is present, is it adequately addressed? | Yes (1)  No (0)  Not reported/ not addressed (0) |  |  |
| If missing data is present, do the authors describe how the missing data was addressed? | Adequately addressed (2)  Poorly addressed (1)  Not addressed/ Not reported (0) |  |  |
