# Supplementary material for: Expression of genes in the skeletal muscle of individuals with cachexia/sarcopenia: A systematic review
Source: PLoS One. 2019 Sep 9;14(9):e0222345. doi: 10.1371/journal.pone.0222345 (PMC6733509; doi:10.1371/journal.pone.0222345)
Supplement: S3 File — (DOCX) [file pone.0222345.s003.docx]

**S3 File. List of 133 Genes Analyzed in Less than Three Research Articles**

| *A2M* | *ADIPOQ* | *AKT1* | *AMBRA1* | *ANGPTL7* | *ANKRD1* | *ATF3* | *ATG5* | *ATG7* | *BCL2 (PI3KCIII)* |
| --- | --- | --- | --- | --- | --- | --- | --- | --- | --- |
| *BEX2* | *BHLHE41* | *CCN1* | *CCND1* | *CDH15 (M-cadherin)* | *CDKN1A (p21)* | *CEBPA* | *CGREF1* | *CHRDL2* | *CHUK (IkKa)* |
| *CKM* | *COMP* | *COX4I1 (COX IV)* | *CS* | *CTSL* | *DEC1* | *DSTN* | *EGR1* | *EPAS1 (HIF2)* | *EVX1* |
| *FARP1* | *FBXO21 (SMART)* | *FGA* | *FGG* | *FIS1* | *FLRT3* | *FST* | *GADD45A* | *GPT* | *GSS (γ-GCS-HS)* |
| *HADHA (HAD)* | *HBA2* | *HBD* | *HDGF* | *HES1* | *HIF1A* | *HIF3A* | *HINT3* | *HK2* | *HLA-F* |
| *HLA-H* | *HOXA11* | *HSD11B1* | *HSP90AB1* | *IER5* | *IGHG1* | *IGLL5* | *IL1B* | *IL32* | *IRX4* |
| *JUN* | *JUND* | *LINC02076 (FLJ35934)* | *LPL* | *MAPK1 (ERK1/2)* | *MAPK8 (JNK1)* | *MAPK14 (p38)* | *MCAT* | *MEF2C* | *MEF2D* |
| *MIR675* | *MMP3* | *MRTFA* | *MRTFB* | *MT-CO3 (COX III)* | *MUSA1* | *MYBPc3 (nMHC)* | *MYC* | *MYH1* | *MYH2* |
| *NDN* | *NDUFA3P3 (LOC644482)* | *NEDD4* | *NFKB1 (NF-kB1/3)* | *NNMT* | *NOS2* | *NR3C1* | *OTUD1* | *PAX3* | *PAX7* |
| *PCK1* | *PCNA* | *PDK4* | *PFKFM (PFK)* | *PINK1* | *PMEPA1* | *PMP22* | *PPARA* | *PPARD* | *PPARG* |
| *PPARGC1A (PGC-1α)* | *PRKN* | *PROX1* | *PRSS36* | *PTPRC* | *RAB10* | *RAB15* | *RCAN1* | *SAA1* | *SERPINA1 (α1-antitrypsin)* |
| *SERPINA5* | *SLC22A3* | *SLC25A37* | *SLC38A1* | *SOD1* | *SOD2* | *SPSB1* | *SRF* | *SUMO2* | *SUMO3* |
| *TFEB* | *TNFRSF12A* | *TRAF6* | *TRIM32* | *UCP2* | *UCP3* | *ULK1* | *UVRAG* | *VEGFA* | *VEGFB* |
| *VEGFC* | *VHL* | *YY1* |  |  |  |  |  |  |  |
