# Supplementary material for: Expression of genes in the skeletal muscle of individuals with cachexia/sarcopenia: A systematic review
Source: PLoS One. 2019 Sep 9;14(9):e0222345. doi: 10.1371/journal.pone.0222345 (PMC6733509; doi:10.1371/journal.pone.0222345)
Supplement: S5 File — (DOC) [file pone.0222345.s005.doc]

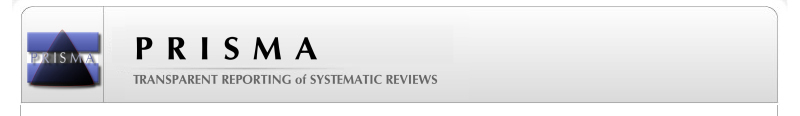
**PRISMA 2009 Flow Diagram**

**Screening**

**Included**

**Eligibility**

**Identification**

Records identified through database searching
(n = 8570 )

Additional records identified through other sources
(n = 0 )

Records after duplicates removed
(n = 6636)

Records screened
(n =6636)

Records excluded
(n =6537)

Full-text articles assessed for eligibility
(n = 99)

Full-text articles excluded, with reasons
(n = 45)

Studies included in qualitative synthesis
(n = 54)

Studies included in quantitative synthesis (meta-analysis)
(n = 0)
